# The Role of Hypoxia and *Cancer Stem Cells* in Renal Cell Carcinoma Pathogenesis

**DOI:** 10.1007/s12015-015-9611-y

**Published:** 2015-07-26

**Authors:** Adam Myszczyszyn, Anna M. Czarnecka, Damian Matak, Lukasz Szymanski, Fei Lian, Anna Kornakiewicz, Ewa Bartnik, Wojciech Kukwa, Claudine Kieda, Cezary Szczylik

**Affiliations:** Department of Oncology with Laboratory of Molecular Oncology, Military Institute of Medicine, Szaserow 128, 04-141 Warsaw, Poland; School of Molecular Medicine, Medical University of Warsaw, Warsaw, Poland; Institute of Genetics and Biotechnology, Faculty of Biology, University of Warsaw, Warsaw, Poland; Emory School of Medicine, Atlanta, GA USA; Department of General Surgery and Transplantology, Medical University of Warsaw, Warsaw, Poland; Institute of Biochemistry and Biophysics, Polish Academy of Sciences, Warsaw, Poland; Department of Otolaryngology, Czerniakowski Hospital, Medical University of Warsaw, Warsaw, Poland; Centre de Biophysique Moléculaire, CNRS UPR 4301, Orléans, France

**Keywords:** Renal cancer, *Cancer stem cells*, Hypoxia-inducible factors (HIF-1α, HIF-2α), von Hippel-Lindau protein (pVHL), Epithelial-to-mesenchymal transition

## Abstract

The *cancer stem cell* (*CSC*) model has recently been approached also in renal cell carcinoma (RCC). A few populations of putative renal tumor-initiating cells (TICs) were identified, but they are indifferently understood; however, the first and most thoroughly investigated are CD105-positive *CSCs*. The article presents a detailed comparison of all renal *CSC*-like populations identified by now as well as their presumable origin. Hypoxic activation of hypoxia-inducible factors (HIFs) contributes to tumor aggressiveness by multiple molecular pathways, including the governance of immature stem cell-like phenotype and related epithelial-to-mesenchymal transition (EMT)/de-differentiation, and, as a result, poor prognosis. Due to intrinsic von Hippel-Lindau protein (pVHL) loss of function, *clear-cell* RCC (ccRCC) develops unique pathological intra-cellular pseudo-hypoxic phenotype with a constant HIF activation, regardless of oxygen level. Despite satisfactory evidence concerning pseudo-hypoxia importance in RCC biology, its influence on putative renal *CSC*-like largely remains unknown. Thus, the article discusses a current knowledge of HIF-1α/2α signaling pathways in the promotion of undifferentiated tumor phenotype in general, including some experimental findings specific for pseudo-hypoxic ccRCC, mostly dependent from HIF-2α oncogenic functions. Existing gaps in understanding both putative renal *CSCs* and their potential connection with hypoxia need to be filled in order to propose breakthrough strategies for RCC treatment.

## Introduction

According to *Cancer Statistics, 2015*, RCC will be responsible for 5 % of all malignancies in men and 4 % in women in the USA. RCC is characterized by a high index of advanced stages at diagnosis (17 % for regional/locally advanced and 16 % for metastatic stage) and a high mortality (5-year survival rate for metastatic disease of only 12 %) [1]. In addition, tumor recurrence occurs in 40 % of patients after curative surgical resection. Moreover, RCC is resistant to chemotherapy and radiation, and weakly sensitive to immunotherapeutic agents such as INF-α and IL-12. But in recent years, the discovery of new molecular and cytogenetic markers has led to the recognition and classification of several novel subtypes of RCC [2] and to the introduction of targeted therapies for metastatic stage RCC, including RTK and mTOR kinase inhibitors [2, 3]. However, complete responses are rare, thus, RCC is still a tumor of unpredictable presentation and poor prognosis [3]. Therefore, future studies have to provide more information concerning molecular and cellular mechanisms underlying RCC development and resistance to targeted therapies.

RCC denotes a set of cancers with diverse genetic and histopathological features, clinical course, and response to therapy. RCC is considered to originate from the highly heterogeneous renal tubular epithelium. Depending on a specific microanatomical site of malignant transformation, a resulting tumor displays unique characteristics, allowing for relatively straightforward identification in most cases [4, 5]. In the Heidelberg classification, ccRCC is the most common histological subtype of RCC [2]. According to the long-standing clonal evolution model, a recent breakthrough multiregional genetic analysis of four RCCs provided evidence of intra-tumor heterogeneity in each tumor, with spatially separated heterogeneous somatic mutations and chromosomal abnormalities (allelic imbalances and ploidy aberrations), that supplies evolutionary reservoir of phenotypic tumor cell diversity through Darwinian selection, predicting therapeutic failure due to adapted aggressive drug-resistant clones of malignant cells. In addition, a single tumor-biopsy specimen was found to contain a non-representative minority of genetic aberrations that are present in an entire tumor, therefore, intra-tumor heterogeneity may explain difficulties in the validation of molecular biomarkers essential in personalized oncology [6]. The novel *CSC* or TIC or TPC model introduces an additional explanation, suggesting that intra-tumor heterogeneity may also result from functional diversity among cancer cells in different states of aberrant differentiation [7, 8]. Although two essential paradigms of cancer propagation – the clonal evolution and *CSC* model – were originally presented as mutually exclusive theories of intra-tumor heterogeneity, they can be easily reconciled and are both an integral part of cancer development and evolution, because in the context of the clonal evolution concept, altered tumor cells with stem cell-like characteristics may well be important units of selection [7–13]. *CSCs* are characterized by an extraordinary capacity for tumor initiation and maintenance due to unlimited self-renewal and multilineage differentiation (multipotency) towards heterogeneous progeny. Possible analogies with normal stem/progenitor cells are still being investigated [8–11].

Following a developmentally hierarchical concept of tumor generation resulting from genetic and/or epigenetic alterations of a very small compartment of normal adult somatic tissue-resident stem/progenitor cells, as described in a number of solid malignancies (breast [14], brain [15], colorectal [16], pancreatic [17], hepatic [18], lung [19], prostate [20], ovarian [21], endometrial cancer [22], malignant melanoma [23], and others), only a few diverse studies, reviewed in chapter 2 and Table 1, have focused on the identification of putative *CSCs* in RCC. These experimental results indicate that different cell subpopulations with stem cell-like properties may be present within this heterogeneous and aggressive tumor. No generally applicable markers are known so far, thus, characterization of putative renal *CSCs* is mainly based on functional studies. What is important, scientists should be aware of the existence of potential multiple, unappreciated and largely unavoidable observational errors in methodology used to study renal TICs. In view of these previously unexplored methodological biases, re-examination of the *CSC* hypothesis in other solid tumors is probably warranted [24].

**Table 1:** In vitro and in vivo properties of various putative *CSC*-like subpopulations identified within RCC of adults

Identification method	Markers			Functional analyses								
	CD105	CXCR-4	DNAJB8	SP				Spheres		ALDH		
Study	Bussolati et al. [47]	Gassenmaier et al. [56]	Nishizawa et al. [57]	Addla et al. [61]	Oates et al. [65]	Huang et al. [66]	Lu et al. [67]	Zhong et al. [58]	Lichner et al. [59]	Debeb et al. [70]	Wang et al. [71]	Ueda et al. [72]^9^
MSC markers (excluding CD105) in vitro	+	+	ND	+^2^	ND	ND	ND	+^4^	+^7^	+^8^	ND	+^10^
MSC marker CD105 in vitro	+	+/−^1^	ND	ND	ND	ND	−	+/−^5^	ND	ND	ND	−
*Stemness* markers in vitro	+	+	ND	+	+	ND	ND	+	+	+	+	+
CD133 marker in vitro	−	−	ND	+/−^3^	ND	ND	ND	+^6^	ND	ND	ND	ND
ALDH activity in vitro	ND	ND	ND	ND	ND	ND	ND	ND	ND	+	+	+
SP in vitro	ND	ND	+	+	+	+	+	+	ND	ND	ND	+
Sphere formation in vitro	+	+	ND	+	ND	ND	ND	+	+	+	ND	+
Clonogenicity in vitro	+	+	ND	+	+	+	+	+	+	+	+	+
Self-renewal in vitro	+	+	ND	+	+	+	ND	+	+	+	+	+
Drug resistance in vitro	ND	+	ND	ND	ND	+	ND	+	ND	ND	ND	+
Radioresistance in vitro	ND	ND	ND	ND	ND	+	+	+	ND	+	ND	+
Tumor initiation (tumorigenicity) in vivo	+	+	+	ND	ND	+	+	+	+	+	+	+
Recapitulation of a tumor of origin (phenocopy) in vivo	+	+	ND	ND	ND	+	ND	+	+	+	+	ND
Generation of serially transplantable tumors in vivo	+	ND	ND	ND	ND	+	ND	ND	ND	+	ND	ND
Endothelial differentiation in vitro and/or in vivo (multipotency) / VM	+	ND	ND	ND	ND	ND	ND	ND	+	ND	ND	ND

*ND* not determined

^1^A major subpopulation within CXCR-4^+^ sphere cells derived only from established cell line SK-RC-17, not from primary cell lines

^2^The CD44 and CD29 MSC markers (however, no significant difference compared to non-SP cells)

^3^Significantly higher cell number in SP than in non-SP cells, however, lower cell number than in the SP of the normal kidney

^4^The CD44 MSC marker, along with CD24 (however, no significant difference compared to non-sphere cells)

^5^Expressed on nearly all non-sphere cells, however, significantly reduced expression on sphere-forming cells

^6^No significant difference compared to non-sphere cells

^7^The CD44 MSC marker, along with CD24

^8^The CD44 MSC marker

^9^Significantly higher ALDH activity in SP than in non-SP cells only in the case of ACHN cell line (ALDH^+^ SP ACHN *CSCs*)

^10^The CD90 MSC marker (however, no significant difference compared to non-SP cells)

Hypoxia, through the activation of HIFs, leads to adaptive changes within a cancer cell, followed by aggressive behavior which contributes to tumor progression and treatment resistance, and, as a consequence, determines an unfavorable prognosis. The correlation of intra-tumor hypoxia with a poor prognosis might be related to the increase in immature, highly aggressive and therapy-resistant malignant cells with stem cell-like characteristics [25–29]. Data reviewed in chapter 3 show this phenomenon for a number of solid tumors, both cell lines and cells directly obtained from patient-derived specimens, but the exact role played by hypoxia in *CSC*-like phenotype promotion began to be appreciated scarcely a few years ago and is still not fully known. In view of its unique pathological intra-cellular pseudo-hypoxic (mimicking hypoxia) phenotype due to a biallelic loss of pVHL function in the absolute majority of clinical cases, with a constitutive HIF-overactivation even in normoxic conditions [2], ccRCC appears as one of the most malignant solid tumors. The exact role of hypoxia in the governance of individual identified putative renal *CSC* populations was not studied, despite quite substantial knowledge concerning HIF activity, especially HIF-2α oncogenic actions, in RCC development and progression. There are also a few findings documenting the hypoxic-induction of HIF-1α-dependent, de-differentiation- and metastasis-associated EMT in RCC. Finally, some putative renal *CSC* markers are activated by hypoxia and possibly contribute to tumor aggressiveness and stem cell functions (see chapter 3 and Fig. 3).

In conclusion, the review analyzes the existing data from both a growing field of *CSCs* and hypoxia, with the emphasis on the most recent studies, and tries to provide a potential, preliminary link between the pseudo-hypoxic and immature *CSC*-like phenotype in RCC (see Fig. 2). Filling gaps in understanding this indifferently studied relationship in RCC is necessary to overcome current treatment limitations by translation of novel discoveries into selective and efficient breakthrough therapies significantly improving prognosis.

## The *CSC* Model in RCC

### CD133^+^ Renal Adult Progenitor Cells

CD133 (prominin-1) is a marker commonly used to define *CSC* populations. It is a five transmembrane domain-glycoprotein, in human first isolated from HSCs, expressed on various types of stem/progenitor cells and differentiated cells, but its biological function is still ambiguous [30]. Two glycosylated renal stem/progenitor cell-associated CD133 epitopes are recognized by monoclonal antibodies – CD133/1 (clone AC133) and CD133/2 (clone 293C3) [31]. Upon *CSC* differentiation, the AC133-specific epitope, but not the entire CD133 protein, is lost [32]. Prominin-1 was investigated as a marker for identification of renal TICs. A very rare population (less than 1 % of total tumor cells) of CD133^+^/CD34^−^ cells was found in human RCC using magnetic bead separation [33]. This population expressed surface markers typical for MSCs [34], such as CD29, CD44 and CD73, the mesodermal marker vimentin, and the embryonic kidney developmental stem cell marker Pax-2 which suggests renal origin. Moreover, CD133^+^ cells could undergo epithelial and endothelial differentiation both in vitro and in vivo. However, they were not able to form carcinomas after subcutaneous injection into SCID mice, indicating no tumorigenic potential [33]. This result is in contrast with the idea that, as in the case of *CSCs* derived from other organs, RCC *CSCs* arise from renal progenitors expressing the CD133 marker [35]. Because of the potential of CD133^+^ cells for in vivo endothelial differentiation in SCID mice, they were suggested to play a pivotal role in tumor vasculogenesis. First, these cells, cultured in the presence of tumor supernatant, differentiated into endothelial cells, suggesting that the tumor microenvironment could be involved in their endothelial commitment. Furthermore, CD133^+^ cells, when co-transplanted with cancer cells at the same ratio as in RCC, significantly enhanced tumor engraftment, growth and vascularization – probably by the production of cancer progression-favoring growth factors [33]. This phenomenon cannot be linked to the cancerous nature of tumor-derived CD133^+^ cells, because the same results were obtained with normal human renal tubular CD133^+^ progenitors [36]. In addition, CD133^+^ RCC cells exhibited the same progenitor phenotype as their normal counterparts, thus, they may represent tumor-infiltrating residents of the normal adult kidney [33]. Normal adult multipotent CD133^+^/CD24^+^ progenitor cell subpopulations were isolated from both tubular epithelium [36–38] and parietal glomerular epithelium of the Bowman’s capsule [38–40] within the renal cortex, whereas in the inner medulla they were detectable in the Henle’s loop and its thin limb segments [41]. Embryonic multipotent CD133^+^/CD24^+^ renal progenitors were also identified [42, 43]. Genomic analysis of normal renal tubular and glomerular multipotent CD133^+^/CD24^+^ progenitor cells revealed no significant differences in gene expression patterns which confirms genetic homogeneity [38].

To sum up, CD133^+^ cells, isolated from RCC, seem to be non-tumorigenic and represent resident adult kidney progenitors which may differentiate into endothelial cells forming new blood vessels in vivo in SCID mice, supporting tumor development [33]. However, a precise function of CD133^+^ progenitors in renal carcinogenesis requires more studies.

### Putative Renal *CSCs*

CD105 (endoglin), a surface transmembrane molecule, acts as a part of the receptor for TGF-β1/3, a pleiotropic cytokine regulating different cellular functions including proliferation, differentiation and migration. Endoglin is a hypoxia-inducible protein expressed abundantly by angiogenic endothelial cells, therefore, it is speculated to be involved in hypoxia-initiated neo-vascularization [44–46]. Bussolati et al. [47] isolated a subpopulation of putative TICs using CD105 magnetic cell sorting of renal carcinomas, independently from a histologic type of origin. In addition, CD105^+^ TICs were found in rhabdoid meningioma [48] and osteosarcoma (the CD44^+^/Stro-1^+^/CD105^+^ population) [49]. CD105^+^ TICs, isolated by Bussolati et al., represented less than 10 % of the tumor. The phenotype of in vitro analyzed CD105^+^ cells revealed several stem cell properties: expression of a set of MSC markers, such as CD44, CD90, CD146, CD73, CD29, and the mesodermal marker vimentin; expression of embryonic stem cell markers, such as Nanog, Oct4, Musashi, Nestin, renal Pax-2, and concurrent lack of differentiative epithelial proteins such as CK; growth in non-adhesive spheres; clonogenic ability; self-renewal ability; differentiation potency (in differentiating conditions) into tumor components carrying specific differentiative epithelial and endothelial markers and lack of stem cell antigens. A low number of CD105^+^ cell clones were able to generate serially transplantable carcinomas in SCID mice containing a small fraction of the undifferentiated CD105^+^ tumorigenic cell population and a lot of differentiated CD105^−^ cells. CD105^−^ cells did not induce tumors, indicating the tumor propagating potential of CD105^+^ cells. CD105^+^ clones, originating from transplanted tumors, displayed the same phenotype as primary clones. Re-established RCCs recapitulated the histological pattern of the heterogeneous tumor of origin, reflecting the differentiation capability of CD105^+^ cells. Evidence of in vivo endothelial differentiation of CD105^+^ clones was confirmed by the human origin of blood vessels after RCC xenotransplantation. On the basis of this, renal TICs are thought to contribute to intra-tumor vascularization [47], therefore, angiogenesis in RCC might be supported not only by endothelial cells recruited from adjacent vessels or circulating CD133^+^ progenitors [33]. In vivo endothelial differentiation was also shown for TICs derived from glioblastoma [50] as well as hepatocellular [51], ovarian [52] and breast carcinoma [53], and seems to be rather a general phenomenon of these cells [35]. It should be noted that in vivo differentiation of TICs into endothelial cells suggests a complex connection between tumor and its microenvironment. Emerging evidence indicates that TICs coordinate tumor promotion (growth, angiogenesis, metastatic diffusion) through their interactions with *niche* by two-directional release of some extracellular factors – providing TIC maintenance on the one hand, and tumor progression on the other. Besides soluble factors, tumor MVs (exosomes) emerged as potential candidates for cell-to-cell communication. MVs may reprogram target cells by transferring various bioactive molecules and initiating epigenetic changes, including membrane receptors, proteins, lipids and RNAs [3]. MVs released from CD105^+^ RCC cells promoted in vitro and in vivo tumor angiogenesis – probably by carrying pro-angiogenic mRNAs and miRNAs that were absent in those derived from CD105^−^ differentiated tumor cells. Additionally, MVs favored lung metastases by inducing lung local expression of pre-metastatic *niche* formation enhancing factors [54]. In subsequent research, CD105^+^*CSC*-derived MVs promoted persistent phenotypical changes in mesenchymal stromal cells supporting tumor progression in vitro (migration, matrix remodeling, angiogenesis and production of growth-stimulating cytokines) and in vivo (proliferation and vascularization) [55].

Gassenmaier et al. [56] used a novel approach to identify putative *CSC* markers in RCC by comparing expression of a number of candidate cell surface proteins in two primary cell lines derived from two tumors with different aggressiveness – a slowly progressing localized and rapidly progressing metastatic stage disease. This was reflected by long and short survival of patients, respectively, as well as by different tumorigenic potential in immunocompromised mice and sphere-forming capacity of analyzed cell lines, suggesting differences in the content of TICs. CXCR-4 was found to be the most reliable *CSC* marker. A *CSC*-like subpopulation expressing CXCR-4 was presented at a higher level (5 % comparing to 0.8 %) in the more aggressive cell line (a metastatic stage), and enriched in tumor spheres derived from both primary cell lines and one established cell line SK-RC-17. CXCR-4^+^*CSCs* vs. CXCR-4^−^ cells had a stronger sphere-forming ability and tumorigenicity. Compared to adherently grown cells, CXCR-4^+^*CSCs* from derived spheres expressed *stemness* proteins (Oct4, Sox2 and Nanog) at elevated levels, had a reduced level of epithelial CK (more immature mesenchymal phenotype), and were more resistant to anti-angiogenic RTK inhibitors Sunitinib, Sorafenib and Pazopanib. Being considered to drive RCC progression and metastasis, a high content of CXCR-4^+^*CSCs*, estimated by the amount of CXCR-4 expression, correlated with a worse prognosis (strong prognostic power for patients without metastases at the time of diagnosis). Importantly, CXCR-4 function was needed for maintenance of this *CSC* population (sphere-forming capacity). The above data may suggest that highly tumorigenic CXCR-4^+^ cells residing within both primary RCC cell lines represent the *CSC*-like population. In contrast to results from Bussolati et al. [47], the CD105 marker was only marginally co-expressed in the CXCR-4^+^ cell population in spheres from both primary cell lines. However, CD105^+^ cells represented a major subpopulation within CXCR-4^+^ sphere cells derived from established RCC cell line SK-RC-17. This may indicate that CD105 is not essential for RCC initiation or marks a rare, highly tumorigenic subpopulation of putative renal *CSCs* only in some primary tumors or established cell lines, thus, is not suitable for the identification of *CSCs* in each experimental approach. On the other hand, expression of CD133 was restricted to a small fraction of cells which number did not increase with tumor sphere formation [56].

In addition, Nishizawa et al. [57], using two RCC cell lines, demonstrated that the overexpression of the HSP40 family member, DNAJB8, increased the percentage of SP cells and enhanced their tumor-initiating ability. Conversely, DNAJB8 attenuation declined the percentage of SP cells and reduced tumorigenicity. This observation indicates that DNAJB8 contributes to *CSC*-like phenotype of RCC and appears as a new antigen of renal *CSCs*.

As the identification of *CSC* selective markers is not always easy, the culture system could be useful for functional selection and enrichment of TICs by exploiting their ability to generate spheres [3]. Cells growing in suspension as tumor spheres in serum-free medium, supplemented with growth factors, were isolated from SK-RC-42 RCC cell line by Zhong et al. [58]. The sphere-forming population showed the *stemness* potential of *CSCs*, in contrast to monolayer adherent cells – higher in vitro levels of several stem cell markers (Oct4, Nanog, β-catenin, BMI), ability for self-renewal, stronger tumorigenicity, and resistance to chemotherapeutic agents and irradiation. Furthermore, the immunophenotype of sphere-forming renal *CSCs* suggests that they may play an important role in the evasion of tumor growth from immune surveillance. In contrast, no significant differences were found between monolayer adherent and sphere-forming cells for expression of CD44, CD24 and CD133 markers. Surprisingly, the CD105 marker was expressed on nearly all non-sphere cells, but its expression on sphere cells was significantly reduced. Therefore, data obtained in this study do not definitively address the issue of a relative frequency of *CSCs* in SK-RC-42 sphere-forming and monolayer adherent cells. In addition, Lichner et al. [59] isolated RCC spheres exhibiting *CSC*-like properties coupled to TGF-β-EMT axis, including clonogenicity, self-renewal capacity, tumorigenicity, the ability to differentiate to cell types of the tumor of origin as well as increased expression of stem cell-related transcription factors (Oct4, Nanog, Klf4 and LIN28).

Another functional approach of renal *CSC* isolation was performed by the identification of cells appearing as the SP [60] from both normal and malignant tubular epithelial cells of the nephron, using cytofluorimetric evaluation of Hoechst 33342 dye uptake [61]. This efflux assay, first developed in the hematopoietic system, allows for the selection of a cell population that rapidly extrudes dyes due to specific membrane transporters typically associated with *stemness* [62]. Addla et al. [61] demonstrated about 4 % of normal kidney cells and about 6 % of RCC cells could be defined as the SP. The SP of RCC exhibited in vitro sphere-forming capacity and enhanced proliferative potential as well as expressed more widely β-catenin, Notch, Sonic Hedgehog and Pax-2 stem cell markers, as compared to non-SP RCC cells. However, RCC SP cells were noted both in G_1_ (most of cells) and G_0_ (quiescent) phase, indicating heterogeneity of this population and suggesting that the terms *SP* and *CSC* perhaps cannot be used interchangeably, but further studies are needed. Indeed, researchers still question the full relationship between SP cells and *CSC*-like phenotype [60]. For example, the SP of glioblastoma multiforme does not necessarily contribute to self-renewal and tumorigenicity related to *CSCs* [63]. These questionable interpretations suggest that the reliability and reproducibility of the SP assay regarding to *CSCs* are the challenge and should be improved [64]. Differential expression of the CD133 marker between the SP of the normal kidney and RCC was found (15 and 3 %, respectively*)* [61]. The SP of the normal kidney expressed the same markers as identified in renal CD133^+^ progenitors by Bussolati et al. [36]. According to the RCC SP, above-mentioned results were confirmed in subsequent research of this group [65]. In addition, Huang et al. [66] identified SP cells of 769P RCC cell line (4.8 %). The 769P SP cell fraction possessed the *CSC*-like characteristics, including the ability for increased proliferation, self-renewal and differentiation as well as strong resistance to chemotherapy (related to the ABCB1 transporter) and radiotherapy. Moreover, Lu et al. [67] identified two SP cell subpopulations within cultured 786-O RCC cell line – Rh123^high^ and Rh123^low^. Rh123^high^ cells were the minority among 786-O cells, exhibited a noticeably higher proliferative activity, long-term differentiation potential and resistance to radiation as well as enhanced colony-forming efficiency and tumorigenic potential, compared to Rh123^low^ cells, thus, may be regarded as a novel *CSC*-like cell population of RCC. However, CD105 marker expression was not observed on the surface of 786-O cells. Furthermore, the characterization of RCC SP cells with the high brilliance synchrotron-FTIR spectroscopy revealed the presence of their various subpopulations with diverse biochemistry, but there is neither evidence of a differential role of particular subpopulations in carcinogenesis, nor were any specific set of markers identified [68].

Based on the utility of high ALDH enzymatic activity for the functional identification of *CSCs* [69], Debeb et al. [70] studied cancer cells with stem-cell like features belonging to the HEK 293 T population. HEK 293 T cells, cultured in vitro as 3D spheres in serum-free stem cell-promoting culture conditions, exhibited *CSC*-like phenotype – they contained the larger CD44^+^/CD24^−^/ALDH^+^ cell population compared to cultured monolayer cells, displayed upregulated stem cell survival signaling, including β-catenin, Notch and survivin, had increased expression of several mesenchymal, pro-metastatic EMT-related genes, and were radiation-resistant. Serial xenografts of HEK 293 T cells into immunocompromised mice yielded aggressive, self-renewing tumors. These findings suggest that HEK 293 T is a valuable cell line to study the plasticity of transformed embryonic cells, thus, representing an important research tool for studying molecular mechanisms in putative renal *CSC* maintenance. Strongly tumorigenic renal ALDH^+^*CSC*-like cells were also identified by Wang et al. [71] by the use of the xenograft model of ACHN (pleural effusion metastasis) and Caki-2 RCC cell lines. Isolated cells displayed several stem cell-like properties in vitro, including clonogenic and self-renewal ability, and increased expression of Oct3/4A, Nanog and Pax-2 stem cell markers. In vivo experiments showed that cells with a high ALDH activity had greater tumorigenicity compared to their counterparts with a low ALDH activity, generating new tumors with as few as 25 cells. In addition, Ueda et al. [72] used the combination of two functional approaches, the SP approach and ALDH enzymatic approach, to identify the *CSC*-like population residing within ACHN and KRC/Y RCC cell lines. SP fractions in ACHN and KRC/Y were 1.4 and 1.7 %, respectively. ACHN SP cells revealed a significantly stronger ALDH activity than non-SP cells (about 33 % and about 15 %, respectively). ALDH^+^ ACHN cells had a stronger sphere-forming and self-renewal ability as well as showed enhanced expression of *CSC*-related genes (*stemness*, drug efflux transporter, anti-apoptotic, angiogenic and EMT-related genes) and higher tumorigenic potential in SCID mice compared to non-SP cells with lower ALDH activity. Importantly, whereas ACHN SP cells showed a stronger sphere-forming ability and drug resistance (INF-α) compared to non-SP cells, the tumorigenic ability was only slightly higher, suggesting that only ALDH^+^ cells within the SP population rather than all SP cells possess real *CSC*-like properties. In the second cell line analyzed, KRC/Y, there was no difference in ALDH expression between SP and non-SP cells. Intriguingly, this study found that while SP cells of KRC/Y cell line contained about five times more CD105^+^ cells than non-SP cells, there were no differences in *CSC*-like properties between SP and non-SP cells, therefore, KRC/Y SP cells lack this characteristics. In turn, within more aggressive ACHN cell line, the CD105 marker was expressed only in a few cells. Thus, these results are in conflict with the findings of Bussolati et al. [47], regarding the CD105^+^*CSC* population, and suggest that CD105 may not be a universal *CSC*-related marker of RCC.

Furthermore, recent studies in pediatric kidney cancer, Wilms’ tumor (nephroblastoma), point to an embryonic renal stem/progenitor initiating cell that fails to differentiate properly and undergoes malignant transformation to a NCAM1^+^/ALDH^+^*CSC* [73].

Taken together, although CD105^+^ cells are the most thoroughly studied *CSC* population of RCC [47, 54, 55], CD105 may not necessarily be a universal marker of renal TICs [56, 58, 67, 72].

### Putative Origin of Renal *CSCs*

The origin of putative renal *CSCs* is still unknown, because to date experimental data seem to be incomplete and discordant. All efforts to isolate and characterize resident renal stem/progenitor cells suffer from numerous technical limitations, being only inferred from some similarities of cell-surface markers which is not always unequivocal [4, 74, 75]. Considering a high complexity of the adult kidney, this organ is likely to harbor various stem/progenitor cell pools with restricted differentiation ability which might be involved in the regeneration of different tissue regions after injury [4]. Over the past few years, several populations of resident multipotent stem cells with mesenchymal characteristics were identified particularly in tubular and glomerular components of the nephron from the adult [76] and embryonic human [77], and rodent kidney [75, 78–81], indicating that renal stem cells may exist. Bruno et al. [76] sorted adult CD146^+^ stem cells from human glomeruli deprived of the Bowman’s capsule, that were positive for MSC surface markers (including CD105), the mesodermal marker vimentin and embryonic stem cell markers (Nanog, Oct4, Musashi, Nestin), and negative for the renal progenitor-specific CD133 marker, epithelial markers (CK and E-cadherin) as well as for hematopoietic (CD45 and CD34) and endothelial (CD31) markers. CD146^+^ MSCs exhibited in vitro capability for clonogenicity, self-renewal and multipotency/bipotency (epithelial and endothelial differentiation). Expression of the renal Pax-2 marker suggests that these cells are a tissue-resident population.

In RCC, tumorigenic multipotent/bipotent *CSC*-like CD105^+^ cells lack expression of CD133 and CD24 markers [47], while they are present in normal adult and embryonic renal epithelial [36–43] as well as in corresponding non-tumorigenic tumor-derived CD105^−^ progenitor cells [33]. Thus, it is suggested that putative renal *CSCs* do not originate from CD133^+^ renal progenitors, but rather from a yet undefined, undifferentiated kidney-resident adult CD105^+^ stem cell population retaining mesenchymal phenotype, possibly that studied by Bruno et al. [76]. This is in contrast with the idea of RCC originating from CD133^+^ cells, as in other tumors [30]. Alternatively, CD133^+^ cells are very few in the non-SP population of normal kidney as well as in SP and non-SP RCC cells, compared to the SP of the normal kidney (however, CD133 was not present on presumable normal kidney stem cells, isolated by Bruno et al. [76]), pointing the loss of the CD133 marker which could be a very early event in stem cell differentiation and possibly in malignant transformation. This finding is also in opposition to the common notion that CD133 is a definitive stem cell marker in solid tumors [61]. In line with this, another well described putative renal *CSC* population, CXCR-4^+^, did not express CD133 [56] as well as marker expression on sphere-forming cells vs. adherent cells was not specific [58]. Moreover, CD133 expression was evaluated on samples of human RCC, using the stem cell-specific anti-human-CD133/1 (AC133) monoclonal antibody recognizing the glycosylation-dependent epitope of prominin-1, but no correlation with pathological changes and affected patient prognosis was found [82]. Additionally, an immunohistochemical study of highly differentiated, non-metastatic and Oct4-negative ccRCC revealed high CD133 expression, suggesting that it is a favorable prognostic marker [83]. In contrast to above-mentioned results, a stable transfection of CD133 into HEK293 cells induced their in vivo tumor-initiating properties [84]. In this scenario, Galleggiante et al. [85] has recently described a ccRCC-derived CD133^+^/CD24^+^/CTR2^+^ cell population. It showed some stem cell-like properties similar to those of CD133^+^/CD24^+^ tubular progenitors from the healthy kidney, including in vitro self-renewal and multipotency, and in vivo angiogenic potential, but no expression of MSC markers. Comparing to their non-neoplastic counterparts, CD133^+^/CD24^+^/CTR2^+^ cells could regenerate tumor in vitro in soft agar (small colonies), were more undifferentiated (expressed higher embryonic stem cell marker levels), and had an individual gene expression profile, in particular, CTR2 was identified as a cell membrane protein involved in cisplatin chemoresistance that could be used to discriminate this population from normal progenitors. Furthermore, a very recent study of Varna et al. [86] indicated the enrichment of tumorigenic CD133/CXCR-4-co-expressing cells, considered *CSCs* by the authors, in perinecrotic vs. perivascular areas of primary metastatic RCCs and their xenografts.

In the kidney, which has lower cell turnover compared to highly proliferating organs such as the gastrointestinal tract and skin [4, 35], the evaluation of putative stem cells is fastidious and their role in tissue repair is less evident. It is important to note that the *CSC* theory is mainly based on functional approaches. As a result, TICs do not necessarily have to be derived from normal tissue stem cells [4]. Alternatively, it could be postulated that kidney tissue lacks a true stem cell compartment [35]. First, renal tubular cells may be suggested to modulate their phenotype towards *stemness* in response to microenvironmental stimuli due to plastic cell phenotype evidenced by staying in G1 phase of the cell cycle (cyclin D1 activity) [87]. In addition, renal tubular epithelium is almost entirely quiescent in the basal state [88], but displays a considerable proliferative/regenerative capacity upon acute renal injury. This step does not involve specialized progenitors or a stem cell pool, but instead requires randomly surviving, terminally differentiated tubular epithelial cells [87, 89–91]. Thus, a mechanism of renal *CSC* generation, different from genetic and/or epigenetic alterations of kidney-resident stem cells, might theoretically occur, implying the de-differentiation process of mature tubular epithelial cells towards immature phenotype [4, 35]. Indeed, terminally differentiated tubular cells were shown to de-differentiate through the EMT process and actively proliferate during tubular repair [92, 93]. In breast cancer cells, the inhibition of E-cadherin, a marker strongly associated with epithelial phenotype, was demonstrated to induce mesenchymal traits and enhance self-renewal ability [94]. During regeneration, tubular re-expression of the kidney-specific marker Pax-2 supports a de-differentiation concept [95]. These observations could serve as the bridge between two models of cancer evolution in RCC – the clonal evolution and *CSC* model – since the de-differentiation process may occur at various stages of malignant transformation (selection of aggressive cancer cell clones) [4]. Taking into consideration a high heterogeneity of epithelial structure and a diverse differentiation state of malignant renal convoluted tubular cells – from stem to mature, through progenitor cells, the resulting renal *CSCs* could display variable, stem cell-like properties and malignancy, depending on which cell type underwent neoplastic transformation [4, 35].

Kidney-homing bone marrow-derived MSCs could be involved in renal repair as an alternative mechanism to resident stem/progenitor cells with mesenchymal characteristics – either through direct engraftment into injured kidney or paracrine/endocrine signaling, including mitogenic, anti-apoptotic, anti-inflammatory and pro-angiogenic effects [74, 77, 96]. Thus, according to several studies on human and on mouse models, CD105^+^*CSCs*, identified in RCC, might originate from transformed CD105^+^ bone marrow-derived MSCs [97–101] or mesenchymal/stromal cells [102], rather than from the kidney itself. In particular, generation of RCC of human recipient cell origin arising in the grafted kidney was reported, suggesting the integration in the grafted kidney of circulating cells originating in the recipient and their subsequent transformation in the graft [100]. Moreover, in vitro and in vivo multipotent differentiation of CD105^+^ TICs is consistent with the ability of mutated bone marrow MSCs to generate both tumor and tumor vasculature cells [103]. Additionally, mesenchymal cells, present in the tumor stromal microenvironment, supposedly undergo neoplastic mutations, epigenetic changes or chromosomal aberrations [104–106]. In contrast to cells forming mesenchymal tumors, renal CD105^+^ TICs expressed Pax-2 and were able to generate in vivo serially transplantable tumors being epithelial cancers (carcinomas) as a tumor of origin. Within those tumors, they gave rise to the CD105^+^ undifferentiated, tumorigenic population and CD105^−^ non-tumorigenic differentiated cells, and did not differentiate into adipocytes and osteocytes [47].

**Fig. 1:**
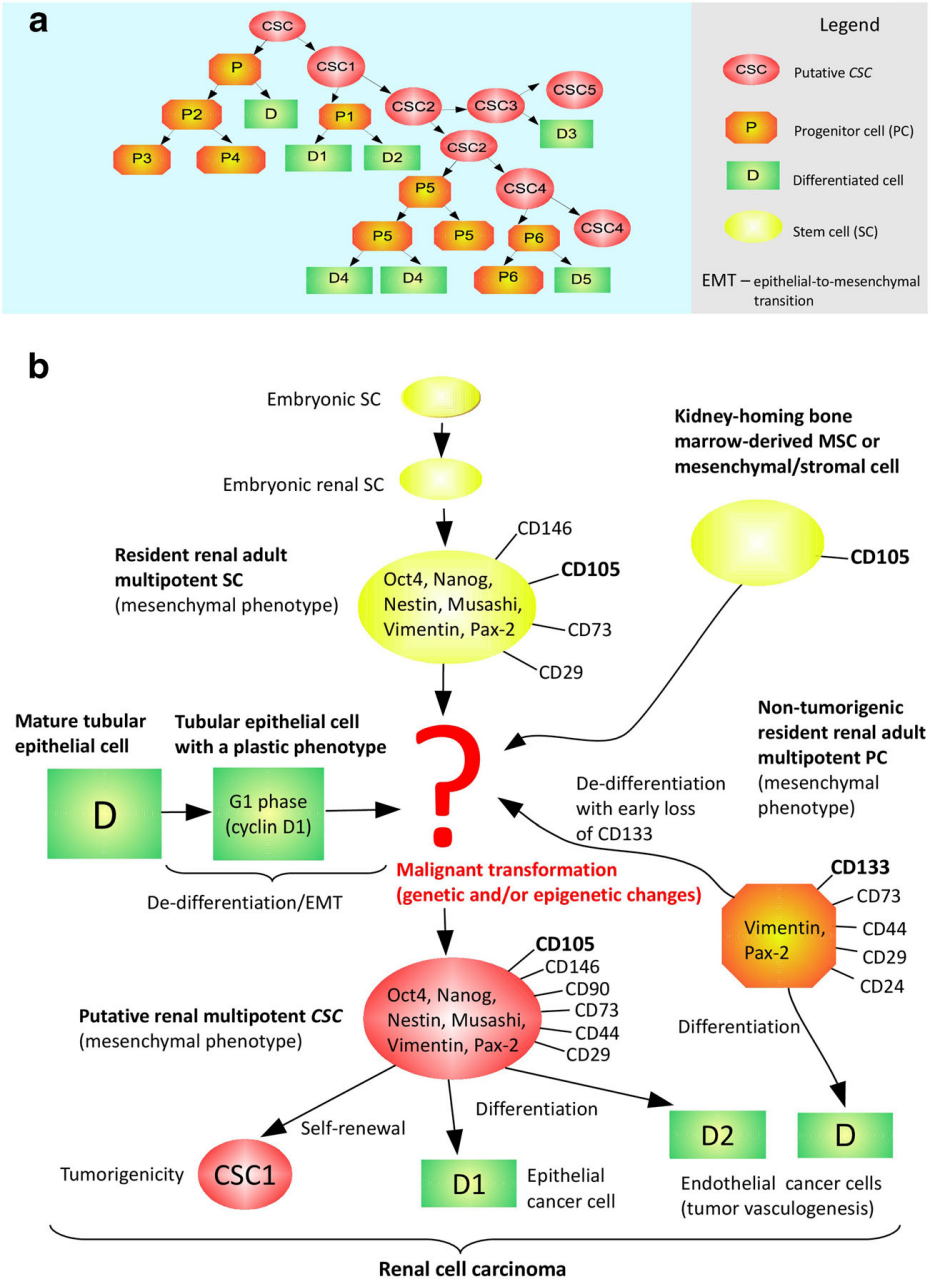
The *CSC* model in RCC. **a**. A general hierarchical model of tumor heterogeneity. **b**. Hypothetical cells of origin of putative renal CD105^+^ TICs. CD105^+^ cells are the first and most thoroughly studied renal *CSC* population identified by now [47]. The most probable cell of origin seems to be a resident renal adult multipotent CD105^+^ stem cell retaining mesenchymal phenotype (a MSC), possibly that studied by Bruno et al. [76]

**Fig. 2:**
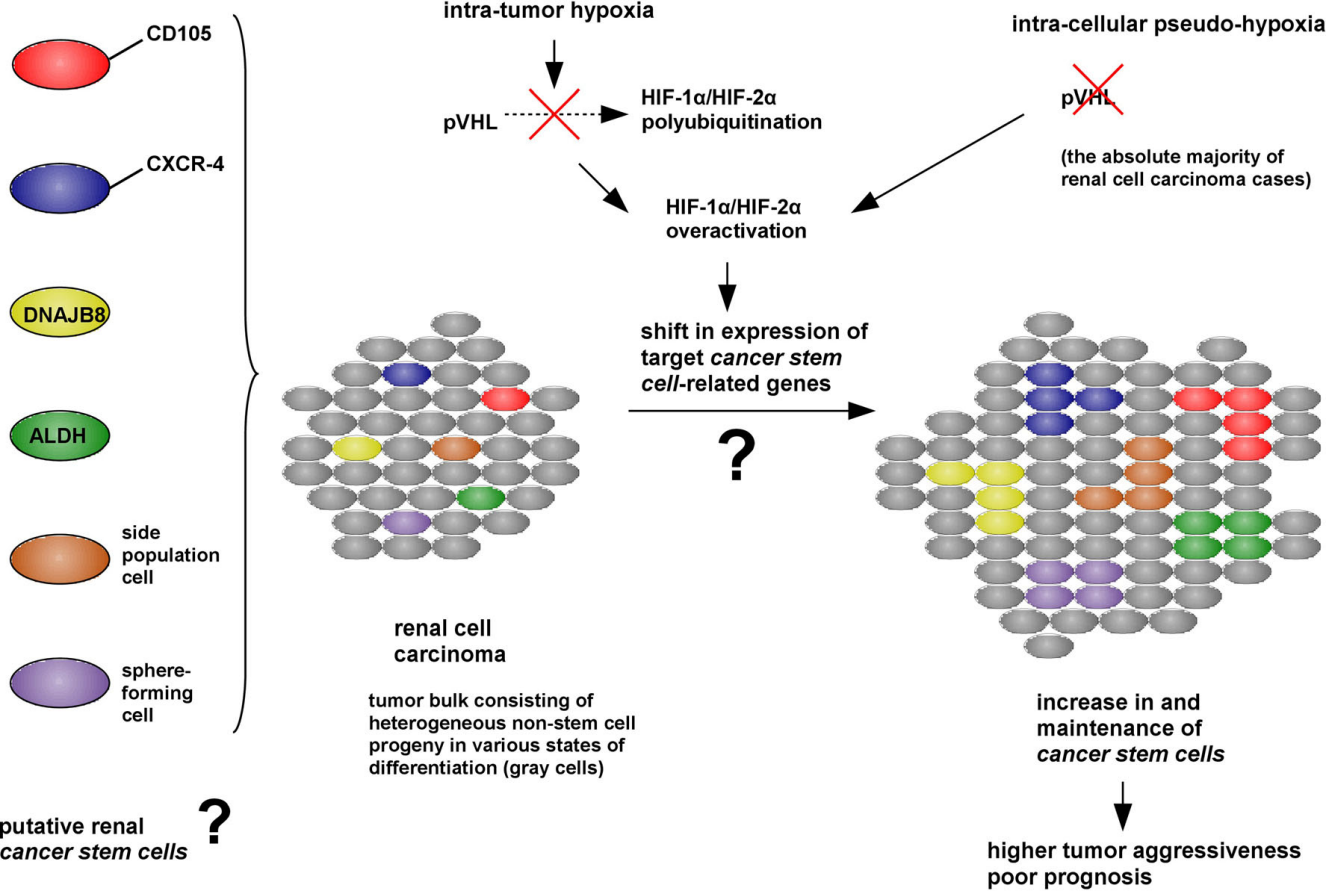
A putative connection between hypoxia and immature *CSC*-like phenotype in RCC

**Fig. 3:**
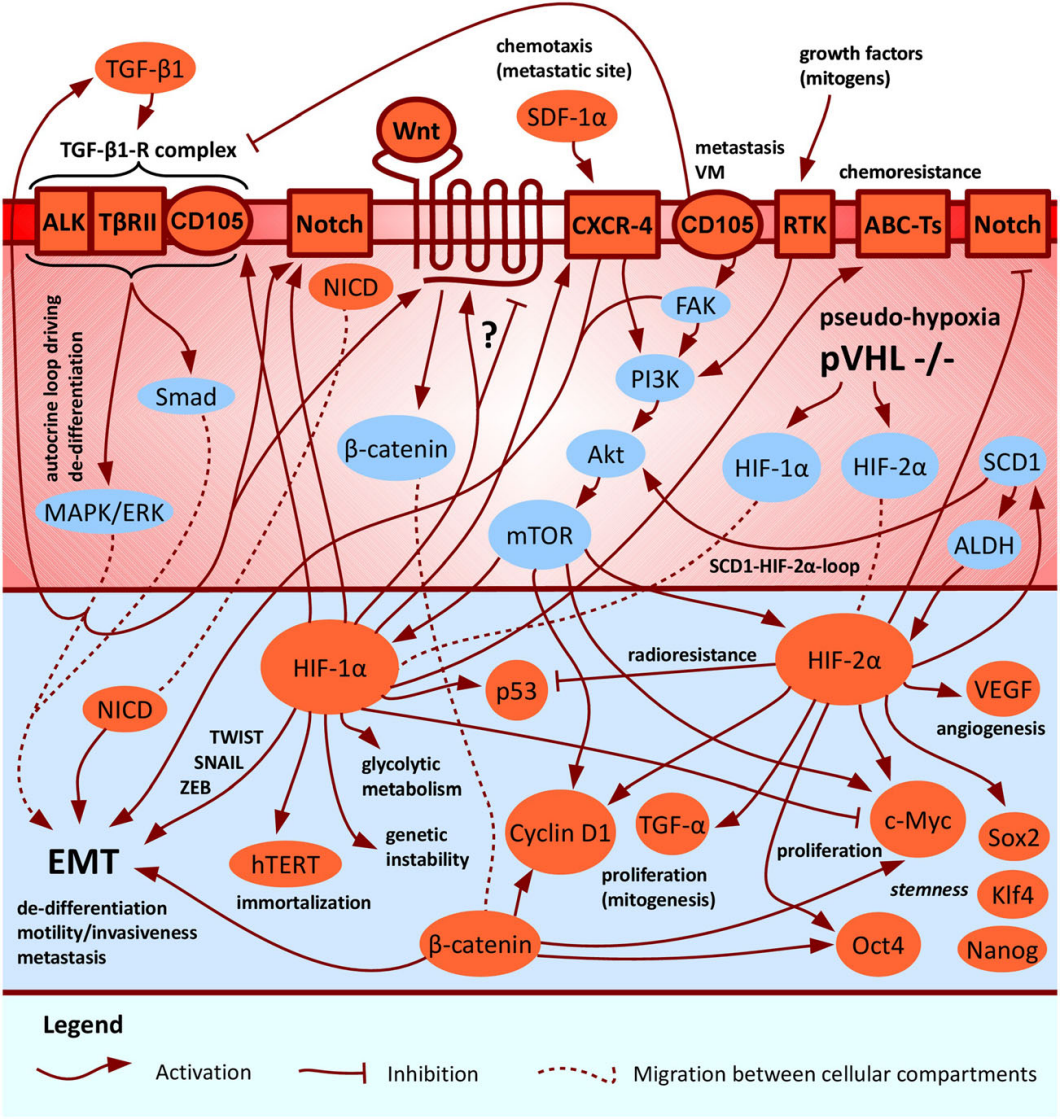
A hypothetical HIF-1α/2α-dependent signaling crosstalk within putative renal *CSCs* involving pathways of three associated markers: CD105, CXCR-4 and ALDH. As a presumable oncogene, HIF-2α is supposed to drive progression of pVHL-defective, pseudo-hypoxic ccRCC (the absolute majority of clinical cases), possibly including promotion of aggressive, immature *CSC*-like phenotype. The figure does not represent pathways in a particular putative renal *CSC* population, but serves as the summary model of all known interactions in various identified populations. A few complements in signaling crosstalk were taken from [25, 168]

In conclusion, the source of the most specifically described putative renal CD105^+^*CSCs* [47, 54, 55], but also of other RCC TICs (see Table 1), may be diverse, not necessarily involving kidney-resident MSCs (see Fig. 1). A common *CSC* marker, CD133, is probably not associated with renal TICs [33, 47, 56, 58, 61, 82, 83], however, some findings contradict this statement [84–86]. Thus, further studies are necessary to eliminate all inconsistencies (Figs. 2 and 3).

## Hypoxia in RCC Progression

### Molecular Regulation of Cell Response to Hypoxia

Tissue hypoxia is defined as reduced oxygen partial pressure ≤ 2 % (≤15 mm Hg). Most mammalian tissues exist naturally at 2–9 % O_2_ (on average 5.5 %, 40 mm Hg) [107], including renal cortex with proximal and distal tubules (7 %, 50 mm Hg). In contrast, the kidney medulla is physiologically poorly oxygenated (1.4–2.8 %, 10–20 mm Hg) [108]. Heterogeneously distributed areas with insufficient blood supply may develop within rapidly growing solid tumors and their metastases. Two types of intra-tumor hypoxia are distinguished – chronic (diffusion-limited, in tumor regions distant from blood vessels) and acute (perfusion-limited, during blood vessel occlusion) [109–111]. Hypoxia compromises cell biology due to impaired energy requiring processes [107]. Cancer cells can survive sustained oxygen deficiency as a result of a coordinated set of adaptive cellular responses to hypoxic stress which are complex processes involving large molecular networks centrally governed by HIFs – HIF-1 and HIF-2 – master regulators of oxygen homeostasis within cells. HIFs are heterodimers composed of an unstable HIF-α (HIF-1α or HIF-2α) subunit, tightly regulated by oxygen (hypoxia-sensitive), and a stable, constitutively expressed (regardless of oxygen level) HIF-β subunit, also known as ARNT [107, 112–117].

pVHL, through its oxygen-dependent posttranslational polyubiquitination of HIF-1α/2α subunits, plays a central role in the mammalian oxygen-sensing pathway [115]. The *VHL* gene was isolated in 1993 [118] and mapped to 3p25–p26. The gene sequence is highly conserved in primates and rodents, and has homologues in *Caenorhabditis elegans* and *Drosophila melanogaster*. Sequence conservation is particularly high across regions known to be involved in pVHL binding to other proteins [119]. pVHL binds to elongin C which forms a multi-subunit complex with elongin B and Cul2/Rbx1 proteins having E3 ubiquitin ligase activity, thus, pVHL is proposed to act as the substrate recognition subunit of this complex recruiting HIF-1α/2α for polyubiquitination resulting in their subsequent 26S proteasome-mediated degradation. The hydroxylation of evolutionarily conserved two proline residues of HIF-α under sufficient oxygen tensions, carried out by members of the EglN prolyl hydroxylase family, also known as PHDs, creates high affinity of modified HIF-1α/2α to the pVHL binding site [113, 120–122]. Classically, HIF-1 and HIF-2 are regulated by rapid degradation of translated HIF-1α and HIF-2α subunits at normal oxygen levels (normoxia) or their stabilization in hypoxia. Decreased oxygen tensions lead to increased activity of HIF. After dimerization of HIF-1α/2α with the HIF-β subunit, constantly present in the cell cytoplasm, HIFs in an active state translocate to the nucleus and bind, in concert with co-activators p300 and CBP, to HREs located in promoters of their hundreds of downstream target hypoxia-inducible genes which become transcriptionally activated [107, 112, 116, 117, 123–125]. Approximately 1–1.5 % of the human genes are regulated by HIFs [126]. A less studied HIF-3α subunit shares a DNA-binding domain with HIF-1α/2α subunits, but lacks a transcription activation domain. Therefore, HIF-3α is considered to serve as a dominant negative regulator of HIF-1α/2α-mediated transcription by the inhibition of the binding of HIF-α subunits to promoters of their target genes [127].

### ccRCC Pathogenesis – Effect of Unique Pseudo-hypoxic Phenotype Due to a Tumor-suppressor pVHL Loss of Function

The genomic 3p25 region, carrying the *VHL* gene, is commonly deleted or altered in ccRCC [128]. The *VHL* gene has a critical *gatekeeper* role in the regulation of growth and differentiation of renal tubular cells. It acts as a classic *two-hit* TSG [119], as defined by Knudson [129]. The loss of normal TSG function due to its biallelic inactivation in renal epithelium may initiate cancer development. As the *hits*, the genome of ccRCC cells generally shows deletion, mutation and methylation within both maternal and paternal loci of the *VHL* gene, in accordance with its role as a TSG [122]. A biallelic loss of function of the *VHL* gene occurs in the majority of sporadic and in all heritable (familial) ccRCC cases [115, 122]. This inactivation is believed to be one of the earliest stages of renal tubular cell neoplastic transformation to ccRCC, as it is observed in pre-malignant cystic lesions of the kidney as well as in advanced tumors [122]. A functional loss of the *VHL* gene usually occurs in ccRCC [115, 122]. The restoration of *VHL* gene function in *VHL −/−* ccRCC cells is sufficient to prevent them from growing as tumors in vivo [130]. In the setting of sporadic ccRCCs, both a first and a second *hit* occur somatically. Thus, they are confined only to somatic cells of an individual, therefore, are not heritable [122]. Somatic mutations of one allele (maternal or paternal) of the *VHL* gene were observed in 42–71 % of sporadic ccRCC cases, accompanied by deletion of the second gene copy, manifested as LOH on chromosome 3p25–p26, in 73–98 % of those lesions. These mutations were heterogeneous in type and distributed across much of the gene [131–135]. A copy of the *VHL* gene can also be epigenetically inactivated (silenced) by hypermethylation of normally unmethylated CpG in the 5′ region (promoter) of the *VHL* gene in about 20 % of sporadic ccRCCs [135, 136]. The absolute majority of those ccRCC cases show methylation of one allele of the *VHL* gene and subsequent deletion of the other (LOH). Thus, DNA methylation may represent another mechanism of the *VHL* gene biallelic inactivation which seems to function identically to inactivating somatic mutations of its one allele in association with a loss of the second allele. Occasionally, both retained alleles are methylated. This methylation phenomenon was detected for a number of TSGs [136]. A loss of normal *VHL* gene function is also common in familial ccRCC associated with rare autosomal dominant VHL disease [116, 119]. Individuals with the VHL disease carry in their germline one *VHL* allele inactivated through mutation (a first *hit*) and one wild-type allele [115]. Pathological changes ensue when the wild-type allele is deleted or somatically inactivated due to hypermethylation or mutation (a second *hit*). In an individual with a germline mutation in one allele of the *VHL* gene, the probability of a second *hit* occurring somatically in the wild-type allele is much higher than the probability of two independent somatic *hits* in a normal individual. This fact explains why ccRCC in VHL patients often develops at earlier age than equivalent sporadic tumors in general population [122].

In ccRCC cells, lack of functional pVHL due to a *VHL* gene loss of function through the biallelic inactivation prevents from HIF-1α and HIF-2α polyubiquitination and subsequent proteasome-mediated degradation, even under normoxic conditions, allowing them to increasingly accumulate and form stable HIF-1/2 heterodimers, what results in their pathological constitutive activation. This unique intra-cellular pseudo-hypoxic phenotype, present as an intrinsic tumor feature, mimics intra-tumor hypoxia [2, 137, 138], contributing to dramatic phenotypic shift in expression of HIF target genes promoting tumor aggressiveness, including angiogenesis, proliferation/mitogenesis, survival/resistance to apoptosis, increased invasiveness and metastatic potential, glycolytic metabolism and extracellular acidosis, hence, determining a poor outcome [107, 111, 112, 117, 125, 139–142].

### Hypoxia Effect on *CSC*-Like Phenotype in RCC – General and Specific Evidence

#### *CSC*-Related Pathways

*CSC*-associated genes are also being considered HIF-target. Both long-standing and emerging data show an effect of hypoxia on the maintenance of normal embryonic and multiple adult stem cell populations [26, 27, 29, 143–147]. The reason why hypoxia stimulates stem cell maintenance is largely unknown, but one attractive hypothesis assumes stem cell localization in the low oxygen microenvironment to reduce DNA damage resulting from ROS-associated genotoxic oxidative stress [29, 148]. Most colon CD133^+^*CSCs* are located in hypoxic *niche* protecting them from chemotherapy [149]. *CSCs* within several brain tumors preferentially locate in hypoxic [150, 151] or pseudo-hypoxic (HIF-2α) perivascular (oxygenated) *niche* [151–155]. Hypoxia promotes self-renewal capability and inhibits differentiation of glioma/glioblastoma *CSCs* [154–156]. Glioma *CSCs*, cultured in the presence of oxygen partial pressure lower than 2 %, display less spontaneous differentiation than when cultured in 21 % oxygen [154]. Reduction in oxygen tension in spheroid cell cultures established from glioma dramatically increases the percentage of cells expressing the CD133 *CSC* marker [155, 157], but also enhances their stem cell-like phenotype (Oct4, Sox2 and Nestin expression) [155]. HIF-2α is believed to play a central role in *CSC* maintenance in hypoxia. Compared to HIF-1α, which is expressed both in glioma/glioblastoma *CSCs* and non-stem cancer cells in hypoxia, HIF-2α has a unique expression pattern – it is highly induced only in *CSCs* [154, 158]. In this line, HIF-2α overexpression in glioblastoma cells promotes *CSC*-like phenotype and significantly increases tumorigenicity [158]. However, both HIF-1α/2α were shown to be critical for *CSC* maintenance, as the knockdown of either HIF-1α or HIF-2α reduced self-renewal capacity, enhanced differentiation, attenuated tumorigenicity and increased apoptosis of glioma/glioblastoma and neuroblastoma TICs, resulting in less aggressive tumor phenotype [153, 154, 156, 158]. Interestingly, HIF-2α is not expressed in normal neural stem cells, making it a very attractive target for *CSC* drug development [154].

Hypoxia was shown to modulate critical, specific signaling pathways, that control the capacity for self-renewal, multipotency/differentiation and proliferation of *CSCs*. Molecular links between HIFs and these key regulatory factors confirm the emerging concept that HIFs not only act in metabolic adaptations of cancer cells, but can also control their immature phenotype. A broad spectrum of activities, influenced by HIFs, overlaps with pathways affected by c-Myc, a crucial oncogenic transcription factor. These interactions can influence tumorigenesis of several cancers [159], including ccRCC. In this tumor, HIF-1α/2α appear to have contrary effects on c-Myc. Whereas HIF-1α counteracts c-Myc pathways in cell cycle arrest (proliferation inhibition) of pVHL-deficient, pseudo-hypoxic ccRCC [160] as well as of colon carcinoma [161], HIF-2α promotes c-Myc activity in pVHL −/− ccRCC [160]. HIF-dependent effects on c-Myc can be used to classify pVHL-defective ccRCC into two subtypes – HIF-2α-expressing subtype (displaying increased c-Myc activity and faster proliferation) and HIF-1α/2α-expressing subtype (displaying neither enhanced nor diminished c-Myc activity due to HIF-1α/2α antagonism) [162]. Analogous to c-Myc, while HIF-1α promotes p53 phosphorylation and subsequent p53-induced apoptosis upon γ-radiation, HIF-2α inhibits them, leading to overall reduction of p53 activity under radiation resulting in radioresistance (a significant feature of *CSCs*). From the clinical point of view, similarly to interactions of HIFs with c-Myc, the ccRCC subtype that expresses the HIF-2α has markedly less phospho-p53 (and develops radioresistance) compared to ccRCC subtype expressing both HIF-1α/2α [163]. To sum up, antagonistic effects of HIF-1α/2α towards c-Myc and p53 appear to influence ccRCC development and progression. In view of *CSCs*, this phenomenon has a great clinical significance pointing to HIF-2α oncogenic function in ccRCC. Another stem cell factor, Oct4, was demonstrated to maintain hypoxic tumor cells in an undifferentiated state towards enhanced progression [164]. As c-Myc [160] and Oct4 [164] activation is HIF-2α-dependent, hypoxic regulation of these two stem cell factors may powerfully impact *CSC* formation from cells expressing HIF-2α. Moreover, Oct-4 and c-Myc, along with Sox2 and Klf4, are required for the formation of iPSCs from adult fibroblasts [165], and that hypoxia enhances the generation of iPSCs [166]. In numerous cancer cell lines, including RCC cell lines, HIFs were demonstrated to induce expression of all iPSC-related markers as well as of Nanog embryonic stem cell marker [167]. Like c-Myc and Oct4, Sox2, Klf4 and Nanog are HIF-2α-dependent [168]. The ability of the hypoxic microenvironment to potentiate the Notch pathway in lung adenocarcinoma [169], malignant melanoma [170] as well as glioma *CSCs* [171] was documented. Notch is a HIF**-**1α**-**target [172], however, recent evidence also indicates a regulative role of HIF-2α as signaling repressor [171]. In addition, Wnt/β-catenin signaling governs *CSC* features in response to hypoxia [173]. In ESCs, this pathway was shown to be a HIF-1α-target [174]. The opposite result was reported for colon carcinoma cells in which HIF-1α inhibited Wnt/β-catenin activity [175]. Thus, it can be suggested that the interaction between HIF-1α and Wnt/β-catenin is functionally distinct in various cell types. Finally, proteins regulating other crucial stem cell functions have been identified as HIF-targets. A gene encoding glycoprotein ABC-Ts, *MDR1*, which confers multidrug resistance on a variety of cancer cells, is a direct HIF-1α-target [176]. In addition, *hTERT*, a gene encoding the enzymatic component of human telomerase that promotes immortalization of *CSCs*, is induced by hypoxia in a HIF-1α dependent manner [177]. Hypoxia (HIF-1α) may also enhance genetic instability of *CSCs*, favoring their undifferentiated state [143]. Furthermore, SCD1, an enzyme in the biosynthesis of monounsaturated fatty acids from saturated fatty acids, is upregulated under hypoxia and modulated by HIF-2α in ccRCC. Upregulation of SCD1 in response to HIF-2α enhanced HIF-2α expression in a positive feedback loop mediated by Akt pathway [178]. Indeed, the Akt-downstream protein, mTOR, can directly activate not only HIF-1α [142], but also HIF-2α [179]. Synergistic effects between SCD1 and HIF-2α contributed to tumor progression: survival (apoptosis inhibition), colony formation ability and migration capacity (metastasis) of pVHL −/− 786-O cells. Once pVHL was restored, SCD1 expression became less sensitive to changes in HIF-2α level [178]. SCD1 is also a key factor for lung spheroid-forming TICs. SCD1 upregulation in lung cancer spheroids is parallel with increased ALDH *CSC* marker activity [180] that was found to be associated with some renal TIC populations [70–72]. It was shown that increased ALDH activity promotes *CSC*-like phenotype of breast cancer through a novel mechanism involving HIF-2α upregulation [181, 182]. Inhibition of ALDH suppressed *CSC* properties both in vitro and in vivo [181]. Importantly, the only study analyzing the influence of hypoxia on putative *CSCs* in RCC was performed on one of identified ALDH^+^ population associated with SP cells (derived from pVHL +/+ ACHN cell line). Hypoxic treatment incresed the number of ALDH^+^ cells 2–3 times; however, more detailed studies were not done [72].

#### De-differentiation/EMT

Beyond evidence for the maintenance of the *CSC*-like potential, hypoxia was indicated to directly alter gene expression of non-stem cell-like neoplastic cells, promoting their spontaneous de-differentiation towards immature stem cell-like phenotype in neuroblastoma [183], breast carcinom*a* [184], glioblastoma [158], ovarian cancer [185] and osteosarcoma cell lines [186]. De-differentiated malignant cells acquired *CSC*-like properties, including self-renewal ability [158, 185, 186], multipotency [186], tumorigenicity [158, 183, 186], invasiveness/metastatic potential [183–186] as well as expression of essential stem cell factors: Notch in neuroblastoma [183], Oct4, c-Myc and Nanog in glioblastoma [158], Oct4 and Sox2 in ovarian cancer [185], and Oct4 Sox2 and Nanog in osteosarcoma studies [186]. In ductal breast carcinoma in situ, tumor cells were less differentiated in hypoxic areas of a malignant lesion, confirming results of de-differentiation experiments on breast carcinoma cell lines [184]. In agreement with these studies, malignant melanoma cell lines may acquire *CSC*-like phenotype through Oct4-mediated cell de-differentiation [187]. There is a strong correlation between tumor clinical stage, differentiation status and behavior. High stage cancers are mostly immature (poorly differentiated) which makes them more aggressive than their more differentiated counterparts [188]. Hypoxia-induced cell de-differentiation contributes to cancer cell plasticity and heterogeneity, hence, has a direct clinical impact. This appears to be a general phenomenon in solid tumors and the mechanism to explain their increased aggressiveness and unfavorable outcome [189].

Molecular mechanisms ruling tumor cell de-differentiation came into focus along with the *CSC* concept. It is noticeable that terms *de-differentiation* and *EMT* show a considerable degree of conceptual overlap. EMT describes how epithelial cancer cells de-differentiate towards mesenchymal, fibroblastoid, spindle-shaped, apolar cells, simultaneously gaining increased motility and invasiveness [188]. EMT endows transitioned cancer cells with stem cell-like properties, providing a mechanistic link between mesenchymal phenotype, *CSCs*, metastatic potential and resistance to therapies [94, 126, 190–195]. An important question in this context is to what degree EMT equates to de-differentiation [188]. A set of diverse extracellular stromal (microenvironmental) signals was reported to induce EMT in malignant epithelial cells, including Wnt/β-catenin and Notch stem cell markers, growth factors such as TGF-β, EGF, FGF and PDGF, transcription factors such as TWIST, SNAIL and ZEB, inflammation cytokines such as tumor necrosis factor-α (TNF-α) and interleukins as well as other specific molecules [191, 194–196]. EMT-triggering mechanisms in cancer are activated by hypoxia [25, 126, 190, 191, 195, 196]. Also hypoxia-responsive EPO, overexpressed especially in ccRCC, might stimulate EMT mediated by PI3K/Akt/mTOR pathway [197].

In pVHL-defective, pseudo-hypoxic ccRCC cells, HIF-1α deregulation was linked to the downregulation of E-cadherin and induction of EMT [198–203] – a second specific hallmark of RCC (beyond abundant angiogenesis) [142]. In addition, hypoxia occurring after renal injury may activate a de-differentiation program in tubular epithelial cells through Oct4 induction providing mesenchymal CD133^+^ progenitor phenotype which may contribute to kidney regeneration. Hypoxia contributes to increased clonogenicity, proliferation and VEGF synthesis by renal medullary progenitors. These effects are reduced by HIF-1α downregulation and followed by progenitor epithelial differentiation [41]. Indeed, CD133 expression is strongly related to the nuclear HIF-1α protein within 786-O RCC cell line and may be upregulated under hypoxic environment [204]. Importantly, with regard to the first and most accurately described population of putative renal *CSCs*, CD105^+^ cells [47], given the endoglin role in activated endothelial cells [44–46], it is not surprising that there is a positive correlation between hypoxia and CD105 protein expression – it is a HIF-1α-target [205]. CD105, along with TβRII and ALK, is a part (a co-receptor) of TGF-β1 receptor complex [44–46, 205, 206]. The complex is characterized by serine-threonine kinase activity [206]. Via MAPK/ERK pathway, which is Smad-independent, TGF-β1 is supposed to crosstalk with Wnt, Notch, IGF and PDGF signaling in order to induce de-differentiation/EMT in osteosarcoma cells. TGF-β1 expression might be activated in the MAPK/ERK-dependent autocrine loop to further promote de-differentiation [186]. Moreover, TGF-β1-EMT axis (Smad-dependent) through TβRII is supposed to play an important role in the in vitro induction of one of sphere-forming *CSC*-like populations in RCC and in associated in vivo sarcomatoid de-differentiation within xenografts derived from these tumor spheres [59]. In line with the activation of TGF-β signaling, endoglin enhances the invasiveness of brain metastatic breast tumor cells by induction of MMPs (involved in metastasis) and chemotaxis to TGF-β from metastatic site [207]. Ewing sarcoma and melanoma research indicates that CD105 can also contribute to tumor aggressiveness independently from TGF-β (inhibition). Endoglin acts in concert with FAK and PI3K signaling to maintain in vitro plastic phenotype and invasiveness (colony- and spheroid-forming ability) as well as in vitro and in vivo growth of tumor cells, which result in poor prognosis [208]. FAK determines tumor aggressiveness by both the induction of matrix MMPs (via PI3K/Akt/mTOR pathway) and EMT [209]. Tumor cell plasticity is an alternative mechanism of tumor blood perfusion to complement the endothelial cell-dependent vasculature. It implies the possibility that some high-grade tumor cells display multipotent embryonic stem cell-like phenotype which enables their differentiation towards abnormal endothelial-like cells forming *de novo* ECM-rich vascular-like channels. This pseudo-tubular structures mimic the pattern of embryonic vascular network, thus, is called VM [208, 210]. The VM phenomenon is induced by hypoxia [208]. The characteristics of undifferentiated cells underlying VM is not well understood [210]. Alternatively, a link between EMT, *CSCs* and VM was proposed. Because tubular-like cells show a significant expression of both endothelial and immature tumor phenotype, VM may represent the transitory step in incomplete *CSC* differentiation into endothelial cells [211, 212]. Indeed, the in vivo ability for endothelial differentiation of CD105^+^ RCC *CSCs* and the consequent involvement in tumor vascularization were described [47]. The presence of *CSC*-dependent vasculogenesis in RCC explains altered embryonic phenotype and behavior of derived endothelial cells such as chromosomal abnormalities and the independence on growth factor supply [212]. In addition, VM was found in xenografts arisen from *CSC*-like spheres of RCC. However, as mentioned above, TGF-β1 signaling, potentially blocked by CD105, plays an important role in the governance of this *CSC* population. Nevertheless, it is unknown if these cells express CD105 [59]. In RCC, VM is an independent unfavorable prognostic factor [213]. In a few studies, in contrast, CD105 was shown to be a tumor repressor by the inhibition of TGF-β signaling [208, 214]. Thus, it is presumed that endoglin function is context-dependent, and TGF-β signaling is highly complex [208]. Finally, another putative renal TIC marker, CXCR-4, is upregulated by HIF-1α in pVHL-defective ccRCC [82, 215, 216]. SDF-1α-CXCR-4 axis can activate EMT/metastasis of *CSCs* [25].

It should be emphasized that intrinsic pVHL-deficient pseudo-hypoxic phenotype is not the common basis of cancer, except the absolute majority of ccRCC clinical cases. Somatic mutations of the *VHL* gene are extremely rare in epithelial solid tumors (including the minority of RCC clinical cases). These pVHL-wild-type tumors often contain chronic and acute hypoxic regions acquired during development. In these regions, active HIF-α subunits accumulate owing to their impaired hydroxylation (lack of oxygen) and can affect tumor cell behavior [142]. HIFs appear to be regulated appropriately within pVHL +/+ RCC cells, meaning that they can respond to hypoxia [124], as in the case of identified putative renal ALDH^+^ ACHN *CSCs* induced by hypoxic treatment [72]. However, it is unknown, what specifically initiates pVHL-wild-type ccRCC [124]. Mathieu et al. [167] showed that both 786-O pVHL −/− cells, cultured in normoxia, as well as pVHL +/+ cells, cultured in 2 % hypoxia, expressed similar levels of Oct4, Sox2 and Nanog markers. The question is, what happens within putative pVHL −/− pseudo-hypoxic renal *CSCs* when acute/chronic hypoxia starts to develop within a tumor? Might pseudo-hypoxic HIF overproduction increase or has it already reached the limit and the number of induced *CSCs* remains at the same level?

Taken together, above-mentioned findings indicate that hypoxia rules immature cancer cell phenotype being an important aspect of the tumor microenvironment which, along with other *niche* components (such as growth factors), plays a fundamental role in conferring higher aggressiveness to tumor cells. It can be assumed that stem and non-stem cell-like populations exist in a dynamic equilibrium within a solid tumor. Therefore, hierarchical models of *CSCs* should be considered to serve as a microenvironment-stimulated interconversion between immature and mature tumor cell components [186]. Hypoxia alters expression level of hundreds of genes and, as a consequence, it is unlikely that a single mechanism can explain cell de-differentiation towards *CSC*-like phenotype induced by reduced oxygen tension. The combined effect of HIF-overactivation may ultimately impose attributes of stem cell identity on more differentiated transformed cells. However, it should be emphasized that this outcome would likely be a rare event – only these hypoxic cells which express a particular level of relevant stem cell factors would be expected to gain *CSC* characteristics [29]. Moreover, it is apparent from current literature that a fraction of cancer cells expressing specific surface marker(s) varies widely between tumors from less than 1 % to upwards of 25 %, but it remains uncertain whether these numbers reflect a true proportion of *CSCs* and/or all marker-positive cells possess *CSC*-like phenotype [126].

### HIF-2α as a RCC Oncogene

It is currently unknown why *VHL* gene inactivation is only linked to ccRCC, although both pVHL and HIFs are expressed in the majority of human cells [124]. It was observed, though, that the medulla of the mammalian kidney is hypoxic in normal physiological conditions [108]. Moreover, among various epithelia tested, renal epithelial cells seem to be particularly sensitive to mitogenic effects of HIF-responsive TGF-α [217, 218]. In addition, hypoxia causes the upregulation of cyclin D1 via HIF activation, stimulating cell proliferation of the renal epithelium, as opposed to other tissues [219–221]. Finally, some differences in proliferation rate between renal and other epithelial cell types have been observed. Intriguingly, epigenetic differences between cells within the kidney and other organs allow the renal epithelium to proliferate under reduced oxygen levels. Thus, it may be concluded that renal epithelial cells, proliferating in hypoxia, are more susceptible to oncogenic effects of the pVHL loss/HIF activation [124]. Other altered genes, which cooperate with the *VHL* gene loss of function to initiate tumor development, should also be identified [115]. Moreover, whether pVHL has any tumor-suppressor functions unrelated to its ability to inhibit HIFs needs to be understood, particularly in view of such pVHL functions as the regulation of ECM formation and turnover, and the modulation of cell death [142].

While both HIF-α subunits show significant homology, most studies were focused on HIF-1α due to its earlier discovery and more universal expression patterns within mammalian tissues than HIF-2α, as HIF-2α expression is restricted to particular cell types, including interstitial cells of the kidney [26, 29]. The role of HIF signaling during kidney development is unclear, but cell type- and stage-specific expression of HIF-1α/2α correlates with expression of critical angiogenic factors such as VEGF and CD105. Although HIF-1α/2α seem to activate hypoxia-responsive genes by similar means, they are non-redundant and regulate both overlapping and unique downstream target genes [138]. HIF-1α/2α were proven to be differently regulated in a number of tumors and seem to have a different impact on tumor behavior and patient outcome in neuroblastoma [222, 223], glioma/glioblastoma [154, 158] as well as breast [224] and non-small cell lung carcinoma [225, 226]. HIF-1α was postulated to be involved in cell adaptation to acute hypoxia, whereas HIF-2α to chronic hypoxia [222, 224]. Different expression patterns and roles of HIF-1α/2α in various tumors indicate the importance of their specificity for cancer survival and propagation [28]. With notable exceptions, HIF-1α itself generally functions as an oncogene in a number of solid tumors, including breast, colon and brain cancer [107, 227], and its levels are informative negative prognostic factors for many malignancies, correlating with worse outcome [111, 228]. In pVHL −/− ccRCC, in contrast, HIF-1α appears to act as a tumor-suppressor [229]. In turn, HIF-2α was supported as a tumor-suppressor in neuroblastoma, despite its promotion of angiogenesis [230]. However, a number of studies indicate that HIF-2α may possibly function as an oncogene, but only in specific tumor types, in certain molecular contexts [152, 224, 225, 231, 232], also in RCC. Several findings on ccRCC show a particularly important role of HIF-2α deregulation in pVHL-deficient pseudo-hypoxic renal carcinogenesis, proving that HIF-2α, not HIF-1α, is an oncogene, at least in the setting of this type of RCC:

1. HIF-1α signaling pathways are activated early in development of VHL disease-associated pre-neoplastic kidney lesions, while HIF-2α is detected in more advanced lesions. The apparent switch from HIF-1α to HIF-2α accumulation in more advanced lesions coincide with increased kidney dysplasia [233]. HIF-2α expression is vital for ccRCC development in a mouse model [234].
2. ccRCC pVHL *−/−* cell lines express either both HIF-1α and HIF-2α, or HIF-2α alone, suggesting that there may be selection pressure to maintain HIF-2α expression or lose HIF-1α expression [121].
3. HIF-2α variants lacking prolyl hydroxylation sites can override tumor suppression by pVHL in animal models, whereas analogous HIF-1α mutants cannot, indicating that HIF-2α inhibition is necessary for pVHL-dependent tumor suppression [234–238].
4. HIF-2α overexpression in ccRCC cell lines expressing functional pVHL results in enhanced xenograft tumor growth [234, 235, 238], in contrast to HIF-1α which inhibits ccRCC growth [237, 238]. Moreover, HIF-2α promotes (and HIF-1α inhibits) pVHL-deficient ccRCC proliferation in vitro via c-Myc signaling [160] and its radiation resistance via phosphorylation inhibition of p53 [163].
5. The elimination of HIF-2α through its siRNA-mediated downregulation is sufficient to suppress the in vivo tumor growth of pVHL-defective ccRCC cells [235, 239].
6. Despite the fact that both HIF-α isoforms target the same HREs during renal carcinogenesis, expression of genes involved in the glycolytic pathway is driven preferentially by HIF-1α [240], whereas in pVHL-defective ccRCC cells, HIF-2α preferentially promotes tumor growth and angiogenesis – it induces expression of cyclin D1, TGF-α and VEGF, which may be understood in the light of HIF-2α being activated mainly during late ccRCC progression, in advanced lesions. However, in pVHL-competent RCC cells and non-RCC cells, cyclin D1, TGF-α and VEGF appear to be potentially responsive to both HIF-α isoforms [238].

## Conclusions, Therapeutic Implications and Future Directions

*CSCs* do not necessarily arise from altered normal stem cells, as they could also originate from normal differentiated cells that acquired self-renewal ability and became malignant [4, 35]. *CSCs* in some tumors may actually not be static and well defined, but rather display dynamic *stemness* properties depending on the microenvironmental context [186]. In line with this, putative TIC populations, identified in RCC, display diverse phenotypes/markers [47, 56–59, 61, 65–67, 70–72], as *CSCs* of malignant melanoma [23] as well as colorectal [16] and ovarian cancer [21]. Because *CSC* model in RCC is mainly based on functional approaches due to lack of generally applicable markers, it is important to optimize isolation and characterization methods of kidney-specific stem/progenitor cells to potentially rule them out as cells of TIC origin [3]. Currently, intensive attention is focused on the hypoxic microenvironment as a central mechanism governing *CSCs* [25–29] and related EMT/de-differentiation [25, 126, 190, 191, 195, 196] which, in turn, are being increasingly recognized as the goal to cure cancer. HIF signaling pathways have been studied in the last few years as extremely attractive targets of therapeutic intervention [117, 140, 241, 242]. Reduction of HIF activity in *CSCs* may promote their differentiation [29]; on the other hand, disturbance of the *CSC niche* may also lead to loss of *stemness* characteristics [243] – both approaches might decrease tumor growth, improve its response to chemo- and radiotherapy, and diminish the ability to relapse. Despite the explosion of information on hypoxia in RCC progression, there are still major questions to be addressed in the field of the dependence of putative renal *CSCs* from their HIF signaling, especially oncogenic HIF-2α.

In light of strong RCC resistance to chemo- and radiotherapy, and its high metastatic index, the identification of cells which are responsible for RCC development and maintenance, coupled with deep knowledge about their biomarkers, intra-cellular pathways and genetic abnormalities, may lead to efficient therapeutic strategies targeting this aggressive population by elimination and/or differentiation in various patient subtypes within different treatment responses and disease stages [3, 244]. A comprehensive molecular analysis of RCC [6, 245, 246] and pharmacogenomics of putative *CSC*s [247] are necessary for development of personalized treatment strategies. Epithelial differentiation of renal CD105^+^ TICs was tested – IL-15 was proposed as an interesting drug candidate [248]. Previous studies provide the rationale for this approach. First, IL-15 was shown in murine studies to be an autocrine survival factor for renal tubular epithelial cells. In addition, in vitro studies indicated that IL-15 upregulates expression of E-cadherin on human renal tubular epithelial cells and inhibits their EMT. Therefore, IL-15 is suggested to act as the sensor of normal tubular homeostasis. It can be speculated that a loss of intra-tumoral secretion of IL-15 by RCC might be a *stemness* protection mechanism of CD105^+^ cells [3]. After IL-15 treatment, renal CD105^+^*CSCs* lacked stem cell markers, expressed epithelial markers and displayed the functional epithelial property of cell polarity. Epithelial differentiated renal *CSCs* were unable to self-renew, non-tumorigenic and drug-sensitive as well as acquired IL-15 secretion guaranteeing a permanent epithelial differentiation state through autocrine loops [248]. A phase I study of intravenous recombinant human IL-15 for the treatment of metastatic RCC was completed in February 2014 (NCT01021059). Another two phase I studies of subcutaneous IL-15 are ongoing (NCT01727076 and NCT02452268). Moreover, endoglin downregulation in epithelial ovarian cancer promoted apoptosis, induced significant DNA damage through modulation of numerous DNA repair genes, and improved platinum sensitivity both in vitro and in vivo, which implies that anti-CD105 therapy would allow dual treatment of both tumor angiogenesis and a subset of aggressive, chemoresistant CD105^+^ tumor cells [249]. A number of strategies to inhibit or downregulate (RNAi) another putative renal *CSC* marker, CXCR-4, have recently been proposed and are undergoing clinical trials [244]. In particular, downregulation of CXCR-4 using siRNA as well as its pharmacological inhibition by AMD3100 (Plerixafor) contributed to sphere formation blockade of CXCR-4^+^ RCC *CSCs*, and increased their responsiveness towards anti-angiogenic agents Sunitinib, Sorafenib and Pazopanib [56]. Also, the DNAJB8 putative *CSC* marker of RCC was evaluated as an immunotherapeutic target in DNA vaccination experiments [57]. Furthermore, honokiol, a multifunctional anti-angiogenic and anti-tumor natural agent [250], inhibits proliferation, reverses EMT (increase in epithelial markers) and suppresses *CSC* properties of RCC cells both in vitro (invasiveness/migration, sphere formation, SP) and in vivo (tumorigenicity) [251]. Honokiol interferes with Ras/Raf/MEK/ERK tumor-promoting pathway [252]. In light of HIF-2α dependence of pVHL-defective ccRCC progression, because SCD1 seems to maintain a positive feedback loop that upregulates HIF-2α towards enhanced tumor progression, therapy targeting both SCD1 and HIF-2α might be effective in the treatment of pVHL −/− ccRCC [178]. Indeed, combinatorial application of SCD1 small molecule inhibitor, A939572, with mTOR inhibitor, Temsirolimus, synergistically attenuated in vitro and in vivo tumor growth in advanced/metastatic ccRCC patients [253]. In addition, in vitro studies on pVHL −/− ccRCC cell lines indicate that Tempol, a stable nitroxide, has potential therapeutic activity against HIF-2α [254].

The identification of the *VHL* gene and understanding of its molecular signaling pathways in renal carcinogenesis played a major role in development of novel targeted therapeutics against metastatic RCC. All these drugs share the ability to block HIF-VEGF-VEGFR and HIF-PDGF-PDGFR axis that plays a significant role in tumor angiogenesis – their use may partially reverse the effect of pVHL loss of function, leading to tumor regression. Among currently licensed systemic targeted agents against advanced RCC, gradually introduced to clinical use since 2006, there are small-molecule multiple RTK inhibitors such as Sunitinib (*SUTENT*, *Pfizer Inc.*), Sorafenib (*Nexavar*, *Bayer HealthCare/Onyx Pharmaceuticals*), Pazopanib (*Votrient*, *GlaxoSmithKline*) and Axitinib (*Inlyta*, *Pfizer*); the neutralizing anti-VEGF humanized monoclonal antibody, Bevacizumab (*Avastin*, *Genentech*), given in combination with IFN-α; and inhibitors of serine-threonine mTOR kinase complexes (mTORC1 and mTORC2), Temsirolimus (*Torisel*, *Pfizer*) and Everolimus (*Afinitor*, *Novartis Pharmaceuticals*) [255, 256]. mTOR is a highly conserved kinase implicated in RCC development, as it is involved in transcriptional regulation of HIF-1α [142] and HIF-2α [179]. Novel targeted therapeutics, used in combination or sequentially, significantly improved outcomes of patients with metastatic RCC, showing higher objective response rate and higher median progression-free survival compared to reference treatment. Results from ongoing/planned clinical trials are expected to help to shape effective RCC future therapeutic possibilities [255, 256]. Despite therapeutic progress, complete and durable responses are rarely noted, necessitating chronic therapy for the majority of RCC patients which is often associated with significant toxicity. In addition, the response to treatment with a specific targeted drug differs between patients, suggesting specific molecular mechanisms promoting individual susceptibility to each agent [255]. Patients who primarily respond to anti-angiogenic targeted treatment often develop acquired (adaptive, evasive) resistance [255, 257–260]. The initial inhibition of tumor growth and prolongation of progression-free survival due to anti-angiogenic therapy are associated with relapse and more invasive metastatic disease [212]. Secondary intra-tumor acute hypoxic condition following anti-angiogenic RCC therapy-associated disruption in vasculature was proposed to promote alternative strategies to support angiogenesis (*angiogenic switch*) such as selection of tumor cell clones independent of VEGF due to multiple molecular and/or cellular mechanisms [255, 257]. Moreover, regarding one of identified presumable ALDH^+^*CSC*-like populations of RCC, anti-angiogenic Sorafenib treatment induced a 2–3 fold increase in a number of ALDH^+^ ACHN cells as compared with normal conditions [72]. Furthermore, a study of tumorigenic CD133/CXCR-4-co-expressing cells, contradicting a general conviction that CD133 is not a marker of renal *CSCs*, showed a promoting effect of Sunitinib-induced hypoxia in tumor perinecrotic areas on the number of these cells, thus, Sunitinib was able to generate resistance to its own therapeutic effect [86]. Indeed, high CXCR-4 expression correlates with poor response to anti-angiogenic treatment (including Sunitinib) in metastatic RCC [56, 261]. In vitro studies revealed that *CSC* increase in breast carcinoma xenografts due to anti-angiogenic treatment (Sunitinib and Bevacizumab) followed by hypoxic conditions is primarily mediated by HIF-1α (EMT induction) [262]. In line with this, xenograft studies showed that hypoxia-activated EMT may be responsible for RCC resistance to Sunitinib [198]. Finally, induction of the VM phenomenon, possibly by the switch from normal to embryonic-like tumor vasculogenesis dependent from endothelial differentiation of *CSCs* [211, 212] which was shown in RCC [47, 59] as well as in hypoxic conditions in breast carcinoma [53] and leukemia [263], may also be responsible for tumor escape from conventional anti-angiogenic therapies. However, VEGFR inhibition by Sunitinib, but not VEGF blockade using Bevacizumab, impaired hypoxia-induced endothelial differentiation of renal CD105^+^*CSCs* in vitro, suggesting a VEGF-independent mechanism [264]. Considering a strict dependence of RCC on angiogenesis for tumor promotion, putative RCC *CSCs* may be speculated to display distinct pro-angiogenic properties [3], especially in light of pVHL inactivation.

In view of development of secondary hypoxia limiting the effectiveness of current therapy, anti-angiogenic agents should perhaps be combined with renal *CSC-*targeting drugs. In addition, an innovative therapeutic perspective might be the use of hyperoxia/hyperbaric oxygen to decrease the number of *CSCs* in resistant metastases (e.g., due to restoration of more differentiated phenotype), resensitizing them [265]. Moreover, a highly challenging issue is counteracting hypoxia-related limitations of anti-angiogenic drugs by tumor vessel normalization (restoration of healthy vasculature) using ITPP [266] and a soluble form of VEGFR-2 [267] in order to inhibit *CSC* selection and improve treatment efficiacy. Another problematic phenomenon for RCC therapy may be EndoMT – the de-differentiation process of endothelial cells to invasive CAFs which is induced by TGF-β [268]. Within the tumor microenvironment, CAFs are supposed to cooperate to tumor aggressiveness by the interaction with and modification of *CSCs* [190]. Finally, in the context of tumor hypoxia, the wide use of rhEPO to treat anaemia in cancer patients, especially ccRCC, is still being discussed because of HIF-induced EPO and EPOR overexpression. On the one hand, binding of exogenous rhEPO with EPOR might prevent or stabilize tumor progression by decreasing hypoxia through increasing oxygenation (erythrocytosis). The alternative argument indicates the possibility of rhEPO/EPOR-mediated initiation of autocrine/paracrine pathways promoting progression [197].

## Abbreviations

*CSC*: *Cancer stem cell*
RCC: Renal cell carcinoma
TIC: Tumor-initiating cell
HIF: Hypoxia-inducible factor
EMT: Epithelial-to-mesenchymal transition
pVHL: von Hippel-Lindau protein
ccRCC: *Clear-cell* renal cell carcinoma
INF-α: Interferon-α
IL: Interleukin
RTK: Receptor tyrosine kinase
mTOR: Mammalian target of rapamycin
TPC: Tumor-propagating cell
HSC: Hematopoietic Stem Cell
MSC: Mesenchymal Stem Cell
Pax-2: Paired box 2
CK: Cytokeratin
SCID: Severe combined immunodeficiency
TGF: Transforming growth factor
MVs: Microvesicles
CXCR-4: CXC chemokine receptor-4
HSP: Heat shock protein
SP: Side population
Rh123: Rhodamine 123
ALDH: Aldehyde dehydrogenase
HEK: Human embryonic kidney
CTR2: Cooper transporter 2
ARNT: Aryl hydrocarbon receptor nuclear translocator
Cul2: Cullin 2
Rbx1: RING-box 1
PHD: Prolyl hydroxylase domain-containing enzyme
CBP: CREB-binding protein
HRE: Hypoxia-responsive element
TSG: Tumor-suppressor gene
LOH: Loss of heterozygosity
CpG: Cytosine-guanine dinucleotide
ROS: Reactive oxygen species
iPSC: Induced Pluripotent Stem Cell
ESC: Embryonic Stem Cell
ABC-Ts: ABC transporter
hTERT: Human telomerase reverse transcriptase
SCD1: Stearoyl-CoA desaturase-1
Akt: Protein kinase B
EGF: Epidermal growth factor
FGF: Fibroblast growth factor
PDGF: Platelet-derived growth factor
TNF-α: Tumor necrosis factor-α
EPO: Erythropoietin
PI3K: Phosphoinositide 3-kinase
VEGF: Vascular endothelial growth factor
TβRII: TGF-β receptor II
ALK: Activin receptor-like kinase
MAPK: Mitogen-activated protein kinase
ERK: Extracellular-signal-regulated kinase
Smad: Mothers against decapentaplegic
IGF: Insulin growth factor
MMPs: Matrix metalloproteinases
FAK: Focal adhesion kinase
ECM: Extracellular matrix
VM: Vasculogenic mimicry
SDF-1α: Chemokine stromal cell-derived factor-1α
VEGFR: VEGF receptor
PDGFR: PDGF receptor
ITPP: Inositol trispyrophosphate
EndoMT: Endothelial-to-mesenchymal transition
CAF: Cancer-associated fibroblast
rhEPO: Recombinant EPO
EPOR: EPO receptor
NICD: Notch intracellular domain
